# Serotonin acts through YAP to promote cell proliferation: mechanism and implication in colorectal cancer progression

**DOI:** 10.1186/s12964-023-01096-2

**Published:** 2023-04-12

**Authors:** Huangfei Yu, Tianyin Qu, Jinlan Yang, Qing Dai

**Affiliations:** 1 Department of Oncology, The First People’s Hospital of Zunyi (The Third Affiliated Hospital of Zunyi Medical University), Zunyi, 563003 Guizhou China; 2 Clinical Cancer Center of Zunyi, The First People’s Hospital of Zunyi (The Third Affiliated Hospital of Zunyi Medical University), Zunyi, 563003 Guizhou China; 3 Scientific Research Center, The First People’s Hospital of Zunyi (The Third Affiliated Hospital of Zunyi Medical University), Zunyi, 563003 Guizhou China

**Keywords:** Serotonin, Serotonin transporter, YAP, Colorectal cancer, Serotonylation

## Abstract

Serotonin, also known as 5-hydroxytryptamine (5-HT), is a key messenger that mediates several central and peripheral functions in the human body. Emerging evidence indicates that serotonin is critical in tumorigenesis, but its role in colorectal cancer remains elusive. Herein, we report that serotonin transporter (SERT) transports serotonin into colorectal cancer cells, enhancing Yes-associated protein (YAP) expression and promoting in vitro and in vivo colon cancer cell growth. Once within the cells, transglutaminase 2 (TG2) mediates RhoA serotonylated and activates RhoA-ROCK1/2 signalling to upregulate YAP expression in SW480 and SW1116 cells. Blocking SERT with citalopram reversed the serotonin-induced YAP expression and cell proliferation, inhibiting serotonin’s effects on tumour formation in mice. Moreover, SERT expression was correlated with YAP in pathological human colorectal cancer samples and the levels of 5-HT were highly significant in the serum of patients with colorectal cancer. Together, our findings suggested that serotonin enters cells via SERT to activate RhoA/ROCK/YAP signalling to promote colon cancer carcinogenesis. Consequently, targeting serotonin-SERT-YAP axis may be a potential therapeutic strategy for colorectal cancer.

**Video abstract**

**Supplementary Information:**

The online version contains supplementary material available at 10.1186/s12964-023-01096-2.

## Introduction

Recently, the biological and therapeutic implications of neural regulation in cancers have drawn considerable interest [1, 2]. For example, in some malignancies, such as pancreatic and prostate cancer, nerves have been shown to infiltrate the tumour microenvironment and actively stimulate cancer cell growth and dissemination [3, 4]. This mechanism involves cancer cells inducing the outgrowth of nerves in the tumour microenvironment by releasing neurotrophic factors. Moreover, in return, nerves liberate neurotransmitters, such as adrenaline [5], acetylcholine (ACh) [6], and others, directly into the vicinity of cancer and stromal cells to activate cancer growth and dissemination. However, the overall significance of neural-associated tumorigenesis remains unclear, especially in colorectal cancers.

Serotonin, also termed 5-hydroxytryptamine (5-HT), is an omnipresent biogenic monoamine expressed in the central nervous system and gastrointestinal tract. Peripheral 5-HT is primarily synthesised by tryptophan hydroxylase-1 (Tph1) in the intestine enterochromaffin cells [7]. 5-HT participates in extensive physiological activities (e.g., platelet aggregation, vascular tone, and intestinal motility) by coupling with the 5-HT receptors (5-HTRs). Seven 5-HTR classes and 13 subtypes exist in mammals, reflecting the diversity and importance of serotonergic functions in the human body [8]. Along with 5-HTRs, SERT, a member of Na^+^-dependent transporter family, also biologically regulates the transportation and function of 5-HT [9]. SERT is responsible for the reuptake of 5-HT released into the presynaptic terminal, terminating the synaptic signal and ensuring replenishment of intracellular 5-HT stores during neurotransmission. SERT’s physiological role in 5-HT signalling is substantiated by the drugs targeting SERT. This approach is used to clinically treat various diseases, such as depression, anxiety, obsessive–compulsive disorder, and neuropathic pain [10, 11].

Numerous studies have revealed that 5-HT plays an essential role in the tumorigenesis of many cancers [12]. 5-HT facilitates tumour growth by directly activating downstream mTOR targets in human hepatocellular cancer [13]. Furthermore, via 5-HT2BR, 5-HT also induces the Warburg effect under metabolic stress, promoting cell growth in pancreatic cancer [14]. In some malignancies, particularly in prostate cancer [15], breast cancer [16], and cholangiocarcinoma [17], 5-HT functions as a self-secreting factor stimulating and maintaining cancer cell proliferation. Even though 5-HT is secreted from intestinal tract mucosa, its exact role in colon carcinogenesis has yet to be fully elucidated. The binding property to different 5-HTRs is a key step for its ability to induce signal transduction and malignant biological effects. Blocking 5-HTR could prevent cancer cell responses to 5-HT signalling, inhibiting tumour growth in various cancers [18]. As a unique transporter protein involved in 5-HT metabolism, SERT may also play a crucial role in tumour growth and progression. Drugs such as Selective Serotonin Reuptake Inhibitors (SSRIs) such as fluoxetine may inhibit SERT’s functioning, thereby suppressing colon cancer cell proliferation [19]. Notably, several epidemiological studies have reported that administering SSRIS to patients with long-term clinical depression greatly reduced their risk of suffering colon cancer [20–22]. This outcome suggests that SERT might also participate in colon cancer tumorigenesis.

Previous studies have reported that the central component of Hippo pathway, YAP, is frequently overexpressed in many human tumours. These studies also confirmed that dysregulated YAP expression is essential for cancer initiation and progression [23]. In colon cancer, YAP overexpression is considered an independent predictor of prognosis and metastasis. Moreover, colon cancers appeared to proliferate when YAP was dysregulated [24]. However, the mechanism underlying abnormal YAP overexpression remains unknown. Hayakawa et al. reported that neurotransmitter ACh could activate YAP expression in human gastric cancer cells via aberrant NGF/Trk and cholinergic signalling, promoting tumour growth [25]. Given the unique nature of neurotransmitters in coordinating crosstalk between nerves and cancer, we hypothesized that 5-HT might also act via activating YAP signalling to promote colon cancer carcinogenesis. Accordingly, we studied the interrelationship between neurotransmitter 5-HT signalling and dysregulated YAP pathway, as well as YAP pathway’s implications in colorectal tumorigenesis.

## Materials and methods

### Cell culture, reagents, and animals

The Chinese Academy of Sciences’ (Shanghai, China) Cell Biology Institute supplied the human colon cell lines SW480 and SW1116. Dulbecco’s Modified Eagle’s Medium (DMEM, high glucose, Gibco, Grand Island, NY, USA) was responsible for culturing all cells and supplied a 10% fetal bovine serum (FBS) (Gibco) in a humidified incubator at 37 °C and 5% CO_2_, respectively. MP Biomedicals (Solon, OH, USA) and Lundbeck (Valby, Denmark) supplied the dextran sulfate sodium (DSS) and citalopram. MCE (Monmouth Junction, NJ, USA) supplied the study’s chemical reagents, except for azoxymethane (AOM) and Sigma (St Louis, MO, USA) supplied the rabbit anti-serotonin antibody, and Cell Signalling Technology (Danvers, MA, USA) supplied all other antibodies. The DaSuo Laboratory Animal Co., LTD (Chengdu, China) supplied C57/BL mice.

### Cell proliferation assay

SW480 and SW1116 cells were seeded in 96-well plates at 1 × 10^3^ cells/well and cultured for 12 h to evaluate the effect of exogenous 5-HT on cell proliferation. After 24 h of serum-free starvation, the cells were treated with a medium containing 5-HT at a range of concentrations for 48 h. Cell viability was monitored using Cell Counting Kit-8 (CCK8, Dojingdo, Kumamoto, Japan) according to the manufacturer’s protocol. Briefly, 10 μL of CCK8 solution was added to each well, and the plates were incubated at 37 °C for 2 h. A microplate reader (Bio-Rad Laboratories, CA, USA) was used to detect the optical densities (ODs) of wells at 450 nm. All experiments were performed in triplicate.

### RNA interference and real-time PCR

This study used the sequences of the small interfering RNAs (siRNAs), and primer pairs are listed in the Additional file 1: Table S1. All siRNAs were designed by GenePharma (Shanghai, China), and the primer pairs were obtained from TSINKE Biological Technology Co., LTD (Chengdu, China). Cells were transfected with siRNA using Lipofectamine 2000 (Invitrogen, Carlsbad, CA, USA) according to the manufacturer's protocol. The knockdown efficiencies were verified by western blot or real-time PCR. To analyse YAP and its target gene in cells stimulated with 5-HT and the knockdown efficiencies of Gα subunits, TRIzol (Invitrogen) was utilized according to the manufacturer’s protocol. The procedure included extracting total RNA and reversing transcribed into cDNA using Reverse Transcriptase M-MLV (Takara, Dalian, China). The SYBR Premix ExTaq kit (Takara) was used to perform real-time PCR. Furthermore, the procedure was performed via iQ5 Real-Time PCR Detection System (Bio-Rad). Lastly, 2-ΔΔCT method was used to model the detected genes relative to that of GAPDH.

### Western blot analysis

Cells were seeded in 6-well plates at 2 × 10^4^ cells/well and starved for 24 h, and then pre-treated with drugs for 2 h before 5-HT stimulation. After 4 h the cells were harvested and lysed with RIPA lysis buffer containing protease and phosphatase inhibitors. Protein extracts were divided equally, separated by 12% SDS-PAGE, and then transferred to nitrocellulose membranes via Bio-Rad semi-dry transfer system. The membranes were blocked in 5% non-fat dried milk for 1 h and then sequentially incubated with the primary antibodies overnight at 4 °C and the DyLight 800 AffiniPure goat anti-rabbit IgG or DyLight 680 AffiniPure goat anti-mouse IgG secondary antibodies (EarthOx, CA, USA) for 2 h at room temperature. Lastly, ODYSSEY Infrared Imaging System (LI-ORBiosciences, Lincoln, NE, USA) visually modelled and quantified the immunoreactive bands.

### Measurement of RhoA activity

About 2 × 10^4^ cells were seeded and cultured as described above, after 4 h with 10 μM 5-HT or 5-HT and citalopram (100 μM) stimulation, cells were harvested and measured the activity of RhoA in SW480 and SW1116. A pulldown assay was used via Rhotekin’s Rho binding domain to assess RhoA activity [26]. A Western blot test was conducted to analyze the precipitated GTP-bound RhoA and total RhoA.

### Coimmunoprecipitation

SW480 and SW1116 cells were seeded at the concentration of 5 × 10^4^/mL in culture dish and then pre-treated with 10 μM 5-HT alone or 10 μM 5-HT combined with 100 μM citalopram for 4 h, the cells were harvested in NETF buffer (100 mM NaCl, 2 mM EGTA, 50 mM Tris–Cl [pH 7.4], and 50 mM NaF) containing 1% Nonidet P-40, 2 mM orthovanadate, protease inhibitors, and a phosphatase inhibitor mixture (Life Technologies, Waltham, MA, USA). The 40 μL of protein A or protein G Sepharose beads were used to preclear the sample, and an anti-RhoA antibody was used to carry out immunoprecipitation reactions. A NETF buffer was first used with Nonidet P-40 (1% w/v) to wash The protein A/G Sepharose-bound immune complexes. The procedure was carried out again without the detergent. The immunoprecipitated pellets were heated at 95 °C for 5 min in 70 μL of the sample for SDS-PAGE and analysed for 5-HT via immunoblotting.

### LC–MS/MS analysis

The concentrations of intracellular-stimulated 5-HT in SW480 and SW1116 cells were analysed according to a prior procedure [27]. Briefly, 5 × 10^6^ cells in 10 mL of medium were plated in 60 cm^2^ culture dishes. After incubation with 10 μM 5-HT for 24 h. To remove exogenous 5-HT contaminates, cell suspensions were obtained and washed with pre-chilled PBS three times. The pellets were then extracted using four times the volume of acetonitrile, and the mixtures were centrifuged at 13,000 rpm for 15 min at 4 ℃; the supernatants (100 μL) were then subjected to analysis. Ultra-high performance liquid chromatography (UPLC) separation was performed on an ACQUITY UPLCTM BEH C18 column (Waters Corporation, Milford, MA, USA). The mobile phases comprised 0.1% formic acid (A) and methanol (B). In succession, isocratic elution was performed with 90% A and 10% B. MS/MS analysis employed the Waters Quattro Premier XE Mass Spectrometer (MS) coupled with an electrospray ionization (ESI) source; Masslynx V 4.1 software was used to control UPLC-MS/MS system. The mass spectrometer was operated in positive ion mode with a source temperature of 110 °C, a cone voltage of 20 V, and a capillary voltage of 2.8 kV. Lastly, selective multi-reaction monitoring in positive ionization mode was used to quantify intracellular 5-HT.

### Immunofluorescence of RhoA and intracellular 5-HT

About 1 × 10^3^ cells/well were seeded in 6-well plates which with a slide at the bottom, after treated with 10 μM 5-HT alone or 10 μM 5-HT combined with 100 μM citalopram for 24 h, taken the slides out and then underwent the following procedure: first, they were fixed with 4% paraformaldehyde for 15 min; second, they were permeabilized with 1% TritonX-100 for 15 min; third, they were blocked with 1% BSA for 30 min; and lastly, they were incubated with primary RhoA (1:1000) and 5-HT (1:5000) antibodies overnight at 4 °C. After being washed three times with PBS, the slides were incubated with FITC- or Alexa Fluor 488-labelled secondary antibodies for 20 min. Finally, the positive staining signals in the cytoplasm were observed by a fluorescence microscope.

### In vivo experiments

Twenty-five adult female C57BL/6 mice were divided into five groups. Colon tumours in mice were induced by AOM and DSS, as previously described [28]. Briefly, 10 mg/kg AOM was intraperitoneally injected into mice, and their normal drinking water was replaced with a 2.5% (wt/vol) DSS solution in the next seven days. This procedure was then repeated every fourteen days for four cycles. Respectively, 10 mg/kg 5-HT was intraperitoneally injected (5-HT group), 20 mg/kg citalopram in normal saline was administered intragastrically (citalopram group), and then both 5-HT and citalopram were administered in combination (combined group). Following the procedure, the mice were sacrificed after 90 days, and their blood serum was collected to quantify 5-HT contents. The tumours were separated from each animal, and their sizes and volumes were measured. Immunohistochemistry (IHC) was performed on the tumours to detect YAP and SERT expression. Animals were bred and housed under pathogen-free conditions. All animal experiments were performed according to the guidelines of Zunyi Medical University’s Institutional Animal Experimental Ethics Committee (approval no. 2019-2-215).

### YAP and SERT expression in human CRC tissues

In colorectal cancer (CRC) cohort, 56 formalin-fixed, paraffin-embedded tissue samples were obtained from the Department of Pathology of the Third Affiliated Hospital of ZMU between August 2021 and October 2022. The clinical pathological parameters were included in the bank, including IHC staining for YAP and SERT, tumour size, and axillary lymph node metastasis status. The WHO classification criteria determined the histological type and clinical stage of tumours. Paraffin-embedded sections (normal and tumour tissues) were immunohistochemically stained for YAP and SERT expression. Briefly, slides were deparaffinized, blocked for nonspecific protein binding, and incubated with either YAP (1:200) or SERT (1:100) primary antibodies for 24 h at 4 °C overnight. These slides were then incubated with the biotinylated secondary antibody for 30 min at room temperature (68–72 °F). The 3,3-diaminobenzidine (DAB) chromogen performed a visualization for 2 min. Replacing the primary antibody with preimmune rabbit serum prepared the negative controls. Then, two independent pathologists scored the patients’ clinical characteristics via YAP and SERT staining.

### Measurement of serotonin

After patients and animals were given serotonin and tryptophan-free diet for 48 h, these anticoagulants-free serum samples were then collected and centrifuged at 1000 g at room temperature for 30 min. The supernatant was collected and also stored at − 80 °C. The 5-HT level in serum was measured via a 5-HT ELISA Kit (Jiancheng, Nanjing, China). This procedure was conducted according to manufacturer’s instructions.

### Statistical analysis

GraphPad Prism software used a one-way analysis of variance (ANOVA) with Tukey’s post-test and the student's t-test. The data were expressed as the mean ± SD. *P* values < 0.05 and < 0.01 were considered statistically significant by the researchers.

## Results

### Serotonin acts by enhancing YAP expression to promote colon cancer cell proliferation

**Fig. 1:**
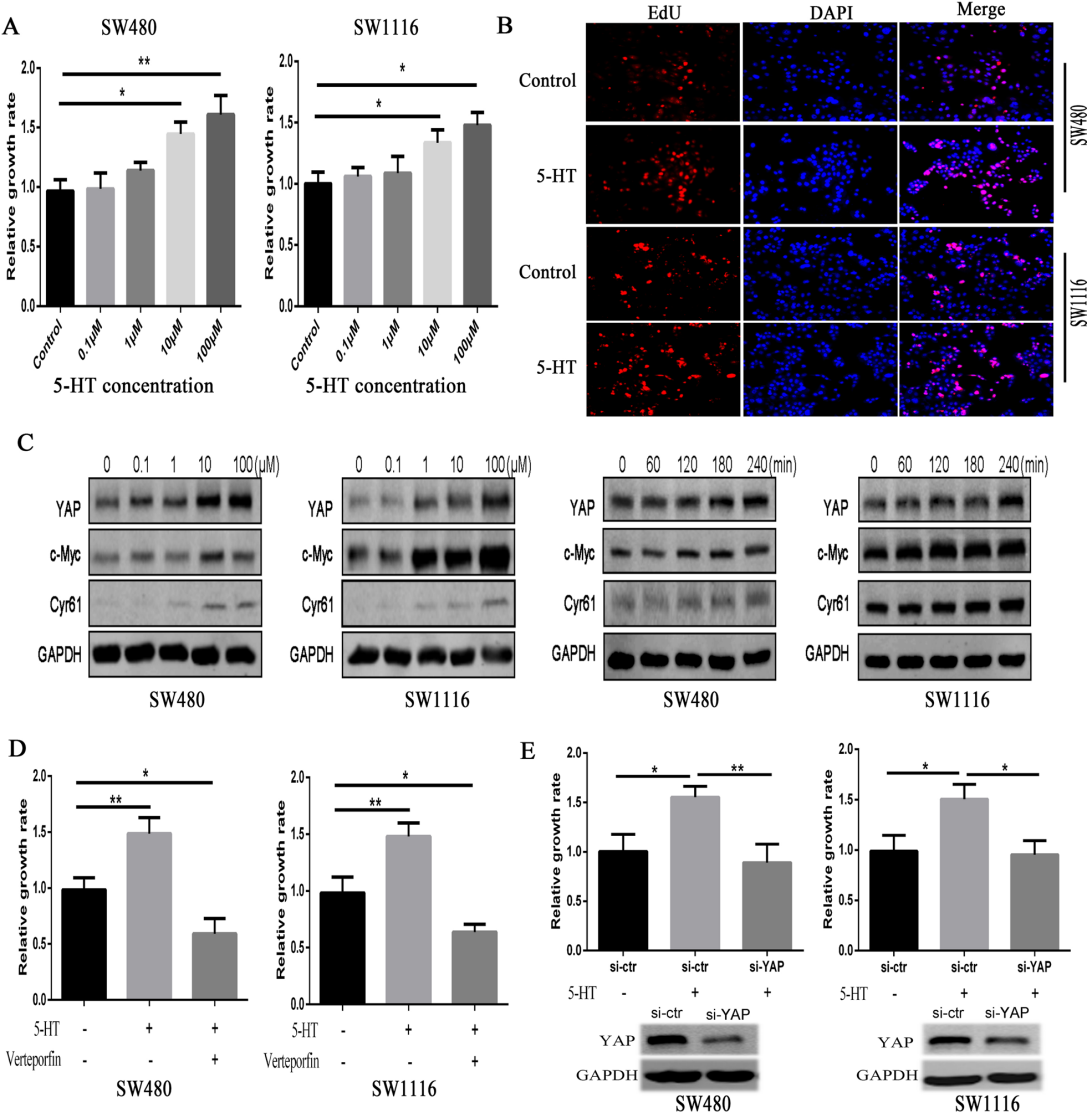
5-HT proliferates colon cancer cells through enhancing YAP expression. **A** 5-HT incubated SW480 and SW1116 cells for 48 h at their indicated concentrations. Furthermore, the CCK-8 assay was used to evaluate the proliferation of cancer cells. *, *P* < 0.05; **, *P* < 0.01. **B** EdU staining (100 ×) determined the** c**ell proliferation of SW480 and SW1116 cells after 10 μM 5-HT stimulation was determined. **C** 5-HT was used to treat the cells at 0.1 μM-100 μM concentrations for 4 h or with 10 μM 5-HT from 30 to 240 min; a western blot test was performed to determine changes in YAP’s expression of its target proteins in SW480 and SW1116 cells. **D** Cells were pre-treated with 1 μM verteporfin 2 h before 10 μM 5-HT stimulation, and 5-HT was combined with a CCK-8 assay to induce proliferation abilities of SW480 and SW1116 cells. *, *P* < 0.05; **, *P* < 0.01. **E** After RNAs were transferred and YAP expression was knocked down in SW480 and SW1116 cells, 5-HT induced cell proliferation was used in combination with CCK-8 assay for this experiment. *, *P* < 0.05; **, *P* < 0.01

To verify the biological effects of serotonin on colon cancer cell proliferation, we performed an in vitro test on two colon cancer cell lines. As shown in Fig. 1A, 10 μM serotonin treatment was administered to SW480 and SW1116, showing cell proliferation after starving the cells for 24 h. Moreover, these proliferative effects were apparent upon 100 μM serotonin stimulation (*P* < 0.05). After serotonin stimulation, an EdU proliferation procedure re-confirmed staining for positive cells (Fig. 1B). Western blot analysis showed that YAP expression, and its downstream proteins c-Myc, Cyr61 in SW480 and SW1116 cells, increased gradually after 4 h of serotonin treatment as the concentrations increased. This analysis confirmed YAP’s expression in a time-dependent manner from 30 min to 4 h, peaking at 4 h under 10 μM serotonin stimulation (Fig. 1C). Moreover, immunofluorescence staining showed that 10 μM 5-HT stimulation for 4 h dramatically increased nuclear YAP expression in both SW480 and SW1116 cells (Additional file 2: Fig. S1).

Because YAP protein has a strong ability to proliferate, many cancers, including colon cancer [29], are frequently viewed as cancer oncopromoter. Given YAP’s effect on cancers, we hypothesized that the enhancement of 5-HT on YAP might be the primary underlying cause of cell proliferation. Our experiment was conducted as follows: first, we used verteporfin to block YAP function. This drug has been proven to prohibit YAP activity from binding its main target transcriptional factor TEAD in the nucleus. Using 5-HT for cell proliferation caused a significant 10 μM verteporfin inhibition (Fig. 1D). Additionally, YAP downregulation restrained colon cancer cell growth (Fig. 1E). Together, these results indicated that 5-HT acted by enhancing YAP expression to promote the proliferation of colon cancer cells in vitro.

### SERT transports extracellular 5HT entry into colon cancer cells

**Fig. 2:**
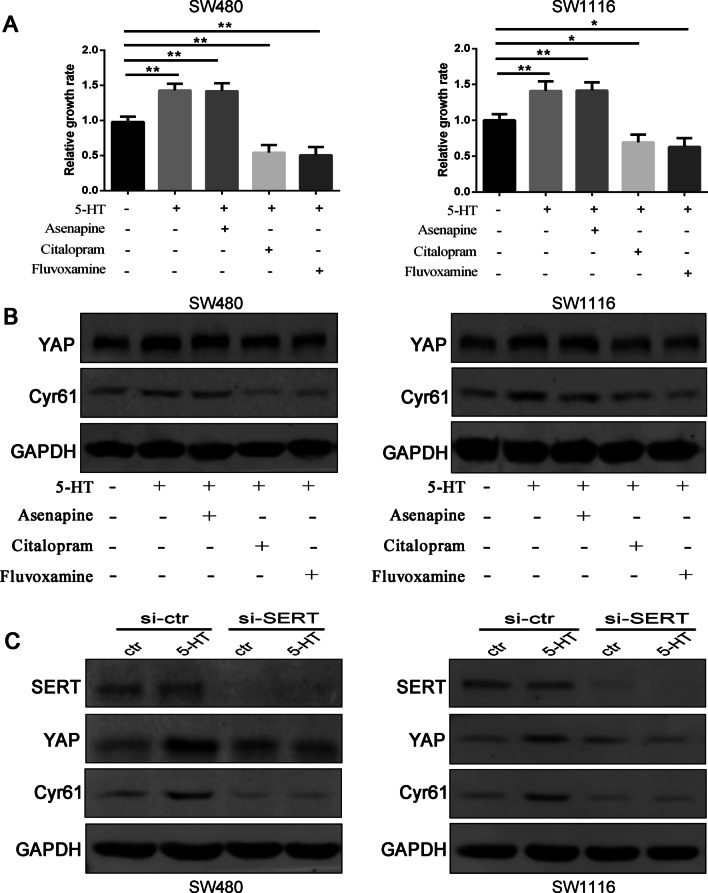
SERT transports extracellular 5HT entry into colon cancer cells. **A** CCK-8 assay treated the cells with the broad spectrum 5-HTR inhibitor asenapine (100 μM) and SERT antagonists citalopram (100 μM) or fluvoxamine (100 μM) for 2 h before 5-HT stimulation. In succession to this procedure, cell proliferation was detected by the CCK-8 assay. *, *P* < 0.05; **, *P* < 0.01. **B** Moreover, a western blot analysis of YAP and Cyr61 expression in SW480 and SW1116 cells were treated with 100 μM asenapine or SSRIs citalopram, and fluvoxamine for 2 h before using 5-HT stimulation. **C** A western blot test was used to stimulate YAP and Cyr61 after knocking down SERT expression in SW480 and SW1116 cells

Both 5-HTRs and SERT, located in the cell membrane (plasma membrane), are functional receptors responsible for transducing 5-HT signals. To determine which receptor primarily mediates 5-HT effects in colon cancer cells, we first pre-treated the colon cancer cells with a 5-HTR broad-spectrum inhibitor (asenapine) and two SSRIs (citalopram and fluvoxamine). This procedure was performed 2 h before adding 10 μM 5-HT. 5-HT treatment combining citalopram and fluvoxamine appeared to reverse the growth of SW480 and SW1116 cells. In contrast, asenapine had nearly no effect on cell proliferation (Fig. 2A). At the protein level, western blot analysis showed that administering 5-HT dramatically inhibited YAP and Cyr61’s expression levels when cells were pre-treated with 100 μM citalopram or 100 μM fluvoxamine. However, their expression was nearly unaltered by asenapine (Fig. 2B). To further exclude the role of 5-HTR in YAP expression, we also targeted the knockdown of four Gα subunits (Gα12, Gα13, Gαq, and Gα11) in colon cancer cells and subsequently stimulated the cells with 10 μM 5-HT. As shown in Additional file 3: Fig. S2A, after downregulating the Gα subunits via siRNA transfection, YAP expressions in SW480 and SW1116 cells were not remarkably altered by 10 μM 5-HT stimulation. However, SERT’s expression knockdown inhibited 5-HT-induced YAP expression (Fig. 2C). Moreover, when hSERT pcDNA3 vector was transferred and SERT was overexpressed in Hela cells, YAP expression was remarkably increased upon 10 μM 5-H stimulation (Additional file 3: Fig. S2B). Consequently, SERT (not 5-HTR) mainly activates YAP expression by transducing 5-HT signals and promoting cell proliferation in the colon cancer cells.

SERT has traditionally been viewed as a transporter of 5-HT into cells. Due to the results above expressing SERT as a primary mediated 5-HT-induced YAP expression and cell proliferation mechanism, we naturally suspected whether these promotional effects in SW480 and SW1116 cells were correlated with the transporting function of SERT. To verify this hypothesis, we treated SW480 and SW1116 cells with a 10 μM 5-HT for 4 h. Then we tested the LC–MS/MS and the intracellular solutes to detect the cytoplasmic 5-HT contents. The prominent dissociative peak (Additional file 4: Fig. S3) appeared at 1.49 min in SW480 and SW1116 cells. This observation was similar to that of the standard one, indicating that SERT was responsible for transporting extracellular 5-HT into the cytoplasm and that 5-HT stimulation resulted in intracellular 5-HT appearing in the cytoplasm of SW480 and SW1116 cells. These data indicated that intracellular 5-HT transported by SERT might mainly mediate YAP upregulation in CRC cells.

### TG2 mediates intracellular 5-HT serotonylates and activates RhoA to promote YAP in colon cancer cells

**Fig. 3:**
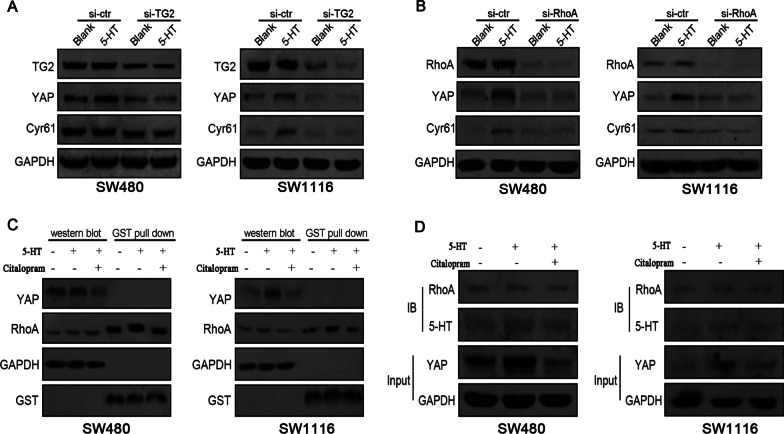
TG2 mediates intracellular 5-HT serotonylates and activates RhoA to promote YAP in colon cancer cells. **A** After siRNA transfection downregulated TG2 expression in SW480 and SW1116 cells, a western blot was used to analyse YAP and Cyr61 expression in cells treated before with 10 μM 5-HT. **B** After downregulating RhoA expression in SW480 and SW1116 cells, YAP and Cyr61 expressions in cells were treated with 10 μM 5-HT. For this procedure, a western blot test modelled these expressions. **C** GST pull-down analysis was used to detect the levels of total and active RhoA in cells stimulated with 5-HT (10 μM) or pre-treated with 100 μM citalopram before 5-HT stimulation. **D** Immunoprecipitation was performed to analyse the expression of RhoA, 5-HT in cells stimulated with 5-HT (10 μM) or pre-treated with 100 μM citalopram before 5-HT stimulation

Intracellular 5-HT is capable of covalently coupling with other proteins under the catalysation of transglutaminase 2 (TG2). In effect, this intracellular modification alters the molecular conformation of the covalented protein and activates it [30]. Proteins of the small G family are the main cellular substrates of TG2. RhoA as a core member of the small GTPase family, has been reported modifies by intracellular 5-HT, leading to it constitutive activity [31]. And the active RhoA has been viewed as an upstream regulator of YAP expression in Hippo pathway [32]. Next, we investigated whether TG2 could internalize 5-HT-stimulated cells with 5-HT as a substrate to regulate YAP expression. Cells were first transfected with TG2 siRNA (siTG2) or controlled with siRNA (siCtrl) for 48 h. Then, these cells were stimulated with 5-HT for 4 h. As displayed in Fig. 3A, 5-HT-induced YAP expression was significantly reversed in SW480 and SW1116 cells upon TG2 knockdown. Next, we used siRNA transfection 48 h before 5-HT stimulation to downregulate RhoA expression in SW480 and SW1116 cells. As a result, RhoA knockdown (Fig. 3B) remarkably reversed 5-HT-induced YAP expression. Next, GST pulldown assays were used to measure RhoA activity in colon cancer cells. Active RhoA expression (Fig. 3C) was dramatically increased in SW480 and SW1116 cells subjected to 10 μM 5-HT treatment. Meanwhile, 100 μM citalopram reversed 5-HT-induced active RhoA expression, suggesting that RhoA was responsible for 5-HT-induced YAP expression in colon cancer cells.

Subsequently, we performed immunoprecipitation experiments to investigate whether RhoA was modified by 5-HT in SW480 and SW1116 cells. The expression of serotonylated RhoA (Fig. 3D) was increased upon stimulation with 10 μM 5-HT, where 100 μM citalopram for 4 h before 5-HT stimulation dramatically reversed this effect. Immunofluorescence analysis also showed that 10 μM 5-HT treatment co-expressed RhoA and intracellular 5-HT in SW480 and SW1116 cells, whereas citalopram remarkably weakened the expression of intracellular 5-HT (Additional file 5: Fig. S4). These results indicated that RhoA was indeed serotonylated and activated by intracellular 5-HT in SW480 and SW1116 cells.

### ROCK1/2 mediates 5-HT-induced YAP expression in colon cancer cells

**Fig. 4:**
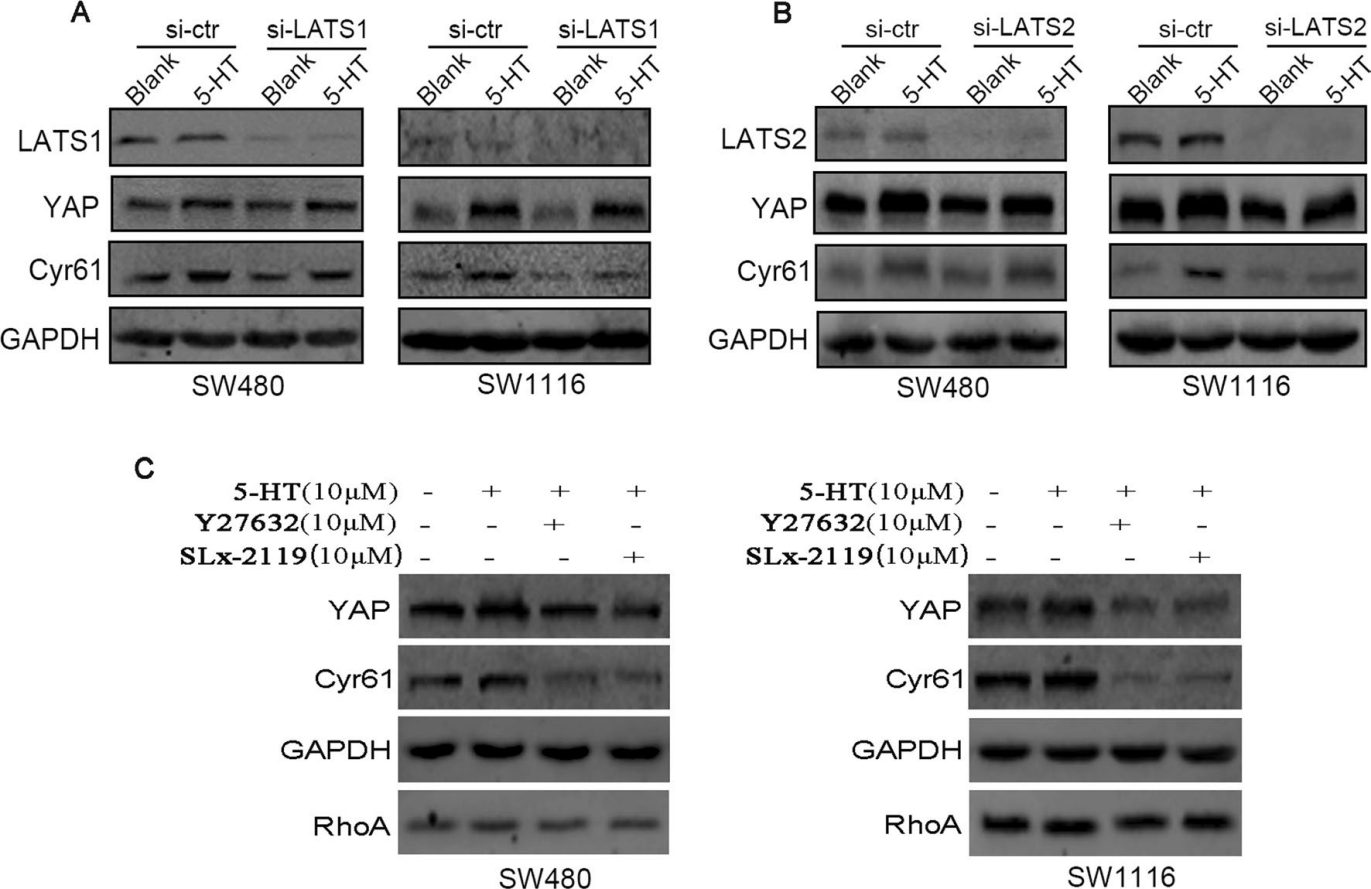
ROCK1/2 but not Lats1/2 mediates 5-HT-induced YAP expression in colon cancer cells. **A** A western blot test was used to analyse YAP and Cyr61 expression in cells treated with 10 μM 5-HT. This procedure was done after Lats1 expression was knocked down in SW480 and SW1116 cells. **B** After Lats2 expression was knocked down in SW480 and SW1116 cells, YAP and Cyr61 expressions in cells treated with 10 μM 5-HT were analysed by western blot. **C** Cells were treated with ROCK1/2 antagonists Y27632 or SLx-2119 (10 μM) for 2 h before 10 μM 5-HT stimulation; a western blot was performed to detect the expression of YAP and Cyr61 in SW480 and SW1116 cells

Lats1/2 and ROCK1/2 regulated YAP expression and phosphorylation [33, 34] via downstream effectors of RhoA. To investigate which molecules participated in 5-HT-induced signalling transduction in colon cancer cells, we first downregulated Lats1/2 expression in SW480 and SW1116 cells by introducing siRNA transfection 48 h before 5-HT stimulation. This procedure had nearly no effect on YAP expression (Fig. 4A,B). When cells were pre-treated with either 10 μM Y27632 (a selective ATP-competitive inhibitor of ROCK1 and ROCK2.) or 10 μM SLx-2119 (a selective inhibitor of ROCK2) for 2 h before 5-HT stimulation, the enhanced YAP expressions in SW480 and SW1116 cells were dramatically reversed (Fig. 4C). This outcome suggested that ROCK1/2 mainly mediated 5-HT-induced YAP expression in SW480 and SW1116 cells.

### 5-HT promotes primary colon carcinogenesis in mice

**Fig. 5:**
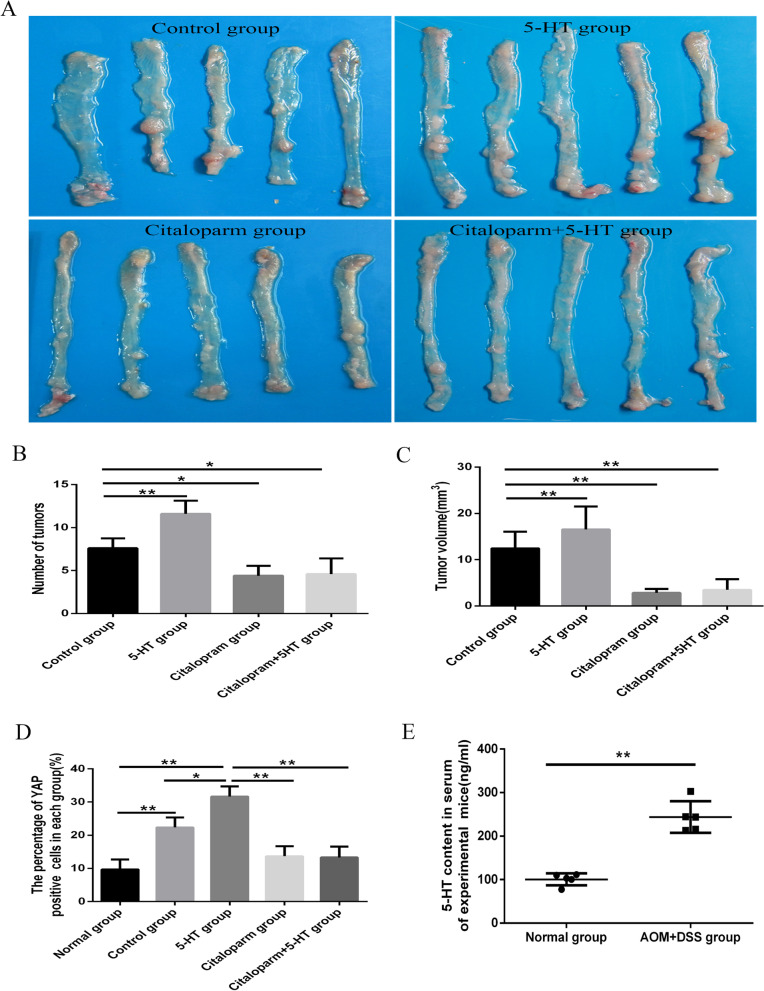
5-HT promotes primary colon carcinogenesis in mice. **A** AOM combined with DSS in indicative groups was used to treat tumour images in the mice’s intestinal tissues. **B** Tumour numbers in the indicated groups of mice. *, *P* < 0.05; **, *P* < 0.01. **C** Tumour volume in the indicated groups of mice. *, *P* < 0.05; **, *P* < 0.01. **D** Immunohistochemistry was performed to analyse YAP expression in colorectal carcinoma tissues of the indicated group of mice. *, *P* < 0.05; **, *P* < 0.01. **E** ELISA analysis detected the serum of the mice’s indicated group’s contents of 5-HT. *,* P* < 0.05; **, *P* < 0.01

An inducible mouse model of colonic cancer was founded using AOM + DSS treatment to investigate the roles of exogenous 5-HT in the carcinogenesis of colon cancer. After 90 days of treatment with 10 mg/kg 5-HT (Fig. 5A), the tumour number and volume in the mice’s intestinal mucosa were 11.60 ± 1.52 and 14.35 ± 2.72 mm^3^, respectively. This outcome showed significantly higher numbers than those of the control group’s mice (7.20 ± 0.84 and 9.86 ± 2.45 mm^3^, respectively). We have also examined citalopram’s effect on the tumorigenesis of colon cancer. The results revealed that citalopram could reduce 5-HT-induced tumour promotion. The tumours in mice and combined groups treated with the citalopram were 4.40 ± 1.14 and 4.60 ± 1.82, respectively. This outcome displayed a significantly lower number than that in the control group (Fig. 5B). The tumour volumes in the citalopram and combined groups were 4.17 ± 1.55 and 4.96 ± 1.83 mm^3^, respectively. This outcome presents a significantly lower number than that in the control group. There were no statistically significant differences in tumour number or volume between combined and citalopram groups (*P* > 0.05, Fig. 5C).

No structural morphology differences in each group’s intestinal mucosa measurement of haematoxylin and eosin (H&E) staining were observed following chemical treatment (Additional file 6: Fig. S5A). YAP, the core effector of Hippo pathway, plays an essential role in CRC’s carcinogenesis. Subsequently, we detected YAP expression in the tissues. The results indicated that the percentage of YAP-positive cells in intestinal tissues from 5-HT group was remarkably higher than that in the control group (31.67 ± 3.06% vs. 22.33 ± 3.41%, *P* < 0.01). The data in citalopram and combined groups were decreased to 13.68 ± 3.08% and 13.33 ± 3.21%, respectively. YAP-positive cells were the lowest among the four groups, measured at 9.67 ± 3.52% (Fig. 5D and Additional file 6: Fig. S5B). Moreover, AOM plus DSS-induced serum 5-HT contents in tumour-bearing mice reached 243.90 ± 41.74 ng/mL. This result was remarkably higher than that in normal mice (100.65 ± 15.92 ng/mL, *P* < 0.05, Fig. 5E). Together, these data suggested that 5-HT promoted tumour growth in colon cancer and, in effect, enhanced YAP expression in colon mucosa. Concurrently, SSRI citalopram prevented colon carcinogenesis in mice.

### Clinical significance of YAP and SERT expression in human colon cancer tissues and high 5-HT content in the serum of CRC patients

**Fig. 6:**
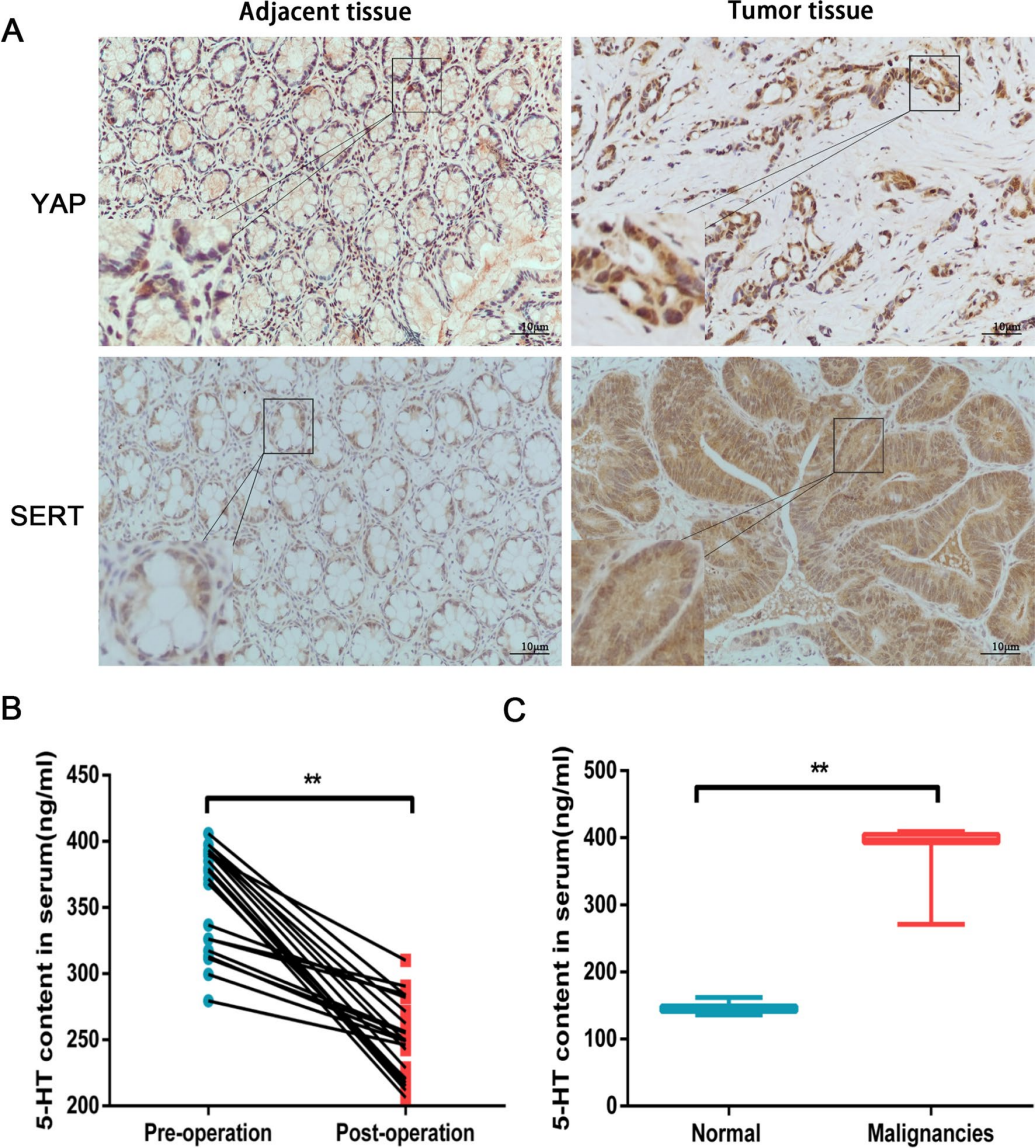
YAP and SERT expression levels are elevated in human colorectal cancer. **A** Immunohistochemistry was used to detect YAP and SERT expression in a representative CRC sample and a matched adjacent normal intestinal tissue (original magnification, 200 ×). **B** ELISA analysis detected the 5-HT content in the serum of patients suffering from CRC before and after surgery. **, *P* < 0.01. **C** ELISA analysis detected 5-HT content in the patients’ serum with multiple metastatic CRC and that of normal people. **, *P* < 0.01

To further determine YAP and SERT’s clinical significance and prevalence in human CRC, we used IHC to assess the expression levels of these proteins in tumour tissue samples from our retrospective cohort of 56 CRC patients after tumour resections (Fig. 6A). Among 56 patients, 43 (76.8%) samples were positive and 13 (23.2%) samples were negative for YAP expression, while 39 (69.6%) samples were positive and 17 (30.4%) samples were negative for SERT expression. YAP and SERT expressions were significantly associated with the lymph node status (both *P* < 0.01), tumour sizes (both *P* < 0.01), and TNM stage (both *P* < 0.01) in colon tumours. However, these expressions did not significantly correlate with gender, tumour location, metastasis, tumour status, or cell differentiation (Table 1). Moreover, YAP and SERT (r = 0.354, *P* < 0.01, Table 2) showed a significant positive correlation, indicating a potential correlation between these proteins in human CRC.

**Table 1:** Clinical correlation of YAP and SERT expression in human colorectal cancer

Clinicopathologic features	Number of patients	YAP positive (%)	*P* value	SERT positive (%)	*P* Value
Age			0.665		0.890
≤ 60	24	21 (87.5)		19 (79.2)	
> 60	32	22 (68.8)		20 (62.5)	
Sex			0.680		0.747
Male	31	27 (87.1)		20 (64.5)	
Female	25	16 (64.0)		19 (76.0)	
Tumour status			0.010*		0.149
T1–2	23	19 (82.6)		18 (78.3)	
T3–4	33	24 (72.7)		21 (63.6)	
Nodal status			0.004**		0.002**
N0	15	7 (46.7)		5 (33.3)	
N1	17	14 (82.4)		13 (76.5)	
N2	24	22 (91.7)		21 (87.5)	
Metastasis			0.314		0.054
M0	50	40 (80.0)		36 (0.72)	
M1	6	3 (50.0)		3 (50.0)	
Tumour location			0.900		0.278
Right	20	16 (80.0)		15 (75.0)	
Left	23	17 (73.9)		16 (69.6)	
Rectum	13	10 (76.9)		8 (61.2)	
Tumour size (cm)			0.025*		0.042*
≤ 3.0	22	14 (63.6)		13 (59.1)	
> 3.0	34	29 (85.3)		26 (76.5)	
TNM stage			0.009**		0.002**
I	4	2 (50.0)		1 (25.0)	
II	11	5 (45.5)		3 (27.3)	
III	35	33 (94.3)		31 (88.6)	
IV	6	3 (50.0)		4 (66.7)	
Differentiation			0.326		0.454
Well	5	2 (40.0)		3 (60.0)	
Moderately	34	25 (73.5)		22 (64.7)	
Poorly	17	16 (94.1)		14 (82.4)	

*P < 0.05; **P < 0.01

**Table 2:** Correlation between the positive staining of YAP and SERT in human colorectal cancer

	YAP	SERT
*Spearman’s rho*		
YAP		
Correlation coefficient	1.000	0.354
Sig. (2-tailed) *P* value		0.008**
N	56	56
SERT		
Correlation coefficient	0.354	1.000
Sig. (2-tailed) *P* value	0.008**	
N	56	56

**Correlation is significant at the 0.01 level (2-tailed)

Furthermore, we used ELISA to detect 5-HT levels in the serum of patients with colorectal cancer. We found that 5-HT content in patients before surgery was (359.05 ± 38.90) ng/mL, while the probability decreased to (250.09 ± 29.32) ng/mL in the patients following their surgery (*P* < 0.01, Fig. 6B). Meanwhile, 5-HT levels in the normal population were (145.96 ± 5.46) ng/mL, whereas its levels in patients with multiple metastatic colorectal cancers were as high as (393.04 ± 28.69) ng/mL (*P* < 0.01, Fig. 6C). These results reveal that 5-HT is highly significant in the serum of patients with colorectal cancer.

## Discussion

As an essential functional molecule in the gastrointestinal tract, 5-HT can regulate gastrointestinal homeostasis via direct innervations of gastrointestinal crypts. However, a consensus on the role of 5-HT in colon cancer development and progression has not been reached. Tutton et al. [35] reported injecting intraperitoneal 5-HT into dimethylhydrazine (DMH)-treated rats significantly promoted colonic tumours, this effect did not influence crypt cell proliferation. Moreover, subsequent studies expand on the proliferative activity of 5-HT in colonic carcinoma [36]. However, 5-HT has been reported to inhibit tumour growth partially because of its restrictive effect on tumour blood flow and immunomodulation of tumour growth [37, 38]. Additionally, 5-HT participates in tumour angiogenesis [39] and gastrointestinal inflammation in mice [40]. Various 5-HTR antagonists and SERT inhibitors appear to suppress colonic tumour growth [41, 42]. These findings are sufficient and ambivalent and elucidate the mechanisms underlying 5-HT’s role in colon cancer. Herein, our data demonstrated that 5-HT acted as a mitogenic factor to facilitate colon cell proliferation and tumour formation. To explore 5-HT as a neurotransmitter in colon cancer development, this process was performed via SERT-RhoA-ROCK1/2-YAP signalling pathway both in vitro and in vivo.

Being a hydrophilic compound, 5-HT cannot permeate cell membranes by diffusion, and it has long been considered a classical extracellular hormone that transduces signals via GPCRs. In our study, SERT, but not 5-HTRs, mainly mediated the increased YAP expression and the proliferation of SW480 and SW1116 cells. This process was done by transporting 5-HT into the cytoplasm. Although we did not identify which 5-HTR subtypes were expressed in these two colon cancer cell lines, we verified that SERT transduced 5-HT signals using SERT siRNAs and SSRI inhibitor citalopram in vitro and in animals. Furthermore, SERT and YAP expression levels were correlated in pathological human CRC tissues. Both displayed clinicopathological features, suggesting that SERT was a key player in mediating 5-HT’s effects on colon carcinogenesis. However, the researchers do not all agree on 5-HTR’s effectiveness in controlling colon cancer. This disagreement might stem from the tremendous heterogeneity in receiving 5-HT signals despite the results in the cell lines mentioned. One or more 5-HTR subtypes presented are responsible for similar malignant biological characteristics in colon cancer, and the same 5-HTR subtype may underlie diverse malignant activities in a single cell line [43, 44]. Consequently, we proposed that 5-HTRs might exert other malignant effects on the tumour progression of SW480 and SW1116 cells, such as metastasis or angiogenesis.

Traditionally, SERT has been considered an intracellular transporter, promoting degradation of 5-HT into the cytoplasm. Nevertheless, various studies have recently shown that intracellular 5-HT initiates additional signalling pathways and induces several biological activities before its metabolic degradation [45]. Notably, 5-HT’s effect in SERT-driven cells is its conjunction with glutamine residues of specific acceptor proteins, such as small GTPases of the Rho and Rab families, in a TG-mediated reaction, culminated in constitutive activation of the targets [31, 46]. This post-translational protein modification was termed ‘serotonylation’ [47]. In addition, serotonylation presents an important molecular event promoting disease progression in many physiological and pathological processes, such as platelet activation [47], insulin secretion [30], and formation of pulmonary arterial hypertension [31]. Histone serotonylation at the site of H3Q5 in neuronal cells exerts a permissive transcriptional activity and sensitives to cellular differentiation [48]. More than 40 intracellular proteins with 50 serotonylation sites at their glutamine residues have been identified in SW480 cells [49], indicating that modifying this post-translational protein significantly affects the development and progression of colorectal cancer.

During serotonylation, TG2 catalyses covalent isopeptide bond formation between the lysine residues’ ε-amino groups and the glutamine residues’ c-carboxamide groups on proteins [30, 46]. In our findings, TG2 regulated intracellular 5-HT to serotonylate and activate RhoA in SW480 and SW1116 cells. RhoA protein’s Gln residues at positions 52, 63, and 136 can be transamidated under TG2 catalysis [50]. RhoA’s Gln-63 transamidation is especially essential for the intrinsic and protein-stimulating of its GTPase activity. Moreover, transamidated 5-HT-induced RhoA’s serotonylation is constitutively active [47]. Active RhoA acts as an upstream regulator to modulate YAP expression via various molecules. This process occurs in Lats-dependent and Lats-independent manners [33, 34]. Our results confirmed the correlation between 5-HT and YAP’s transcriptional activity: 5-HT stimulation increased YAP’s transcriptional activity and accelerated its nuclear localization in SW480 and SW1116 cells. The pharmacological inhibition of Rho-associated protein kinase (ROCK) proteins with Y27632 or SLx-2119 reversed 5-HT-induced YAP expression. This outcome confirmed RhoA/ROCK’s critical effect on its signalling axis in YAP-mediated regulation of 5-HT in colon cancer cells. RhoA-ROCK signalling appears to control YAP expression to maintain intestinal homeostasis and stem cell regeneration in the small intestinal epithelium [51]. Hyperactivate RhoA/ROCK1/actomyosin signalling drives oncogenic TEAD/YAP transcription, a frequent mechanism activating RhoA function in human malignancies [52].

Notably, we found that citalopram may inhibit CRC cell proliferation in vitro and the tumorigenesis of colon cancer in vivo, as well as the content of 5-HT, is abnormally highly expressed in the serum of no matter tumour-bearing mice and patients with colorectal cancer. While SSRIs have been widely used as a first-line drug in clinics for patients suffering some mental disorders (e.g., depression), several epidemiological data confirmed that SSRI treatment might decrease colorectal cancer in depressed patients [20–22]. Despite a depression diagnosis, these results were not conclusively associated with cancer risk [53]. However, increasing evidence indicated that a bad prognosis may cause depression increasing the mortality rate in the cancer population overall [54]. In sum, we provided some new evidence on SSRI administration aiming to prevent and treat colorectal cancer, especially in patients with depression. However, further clinical investigations should be conducted to validate this novel strategy in CRC patients.

**Fig. 7:**
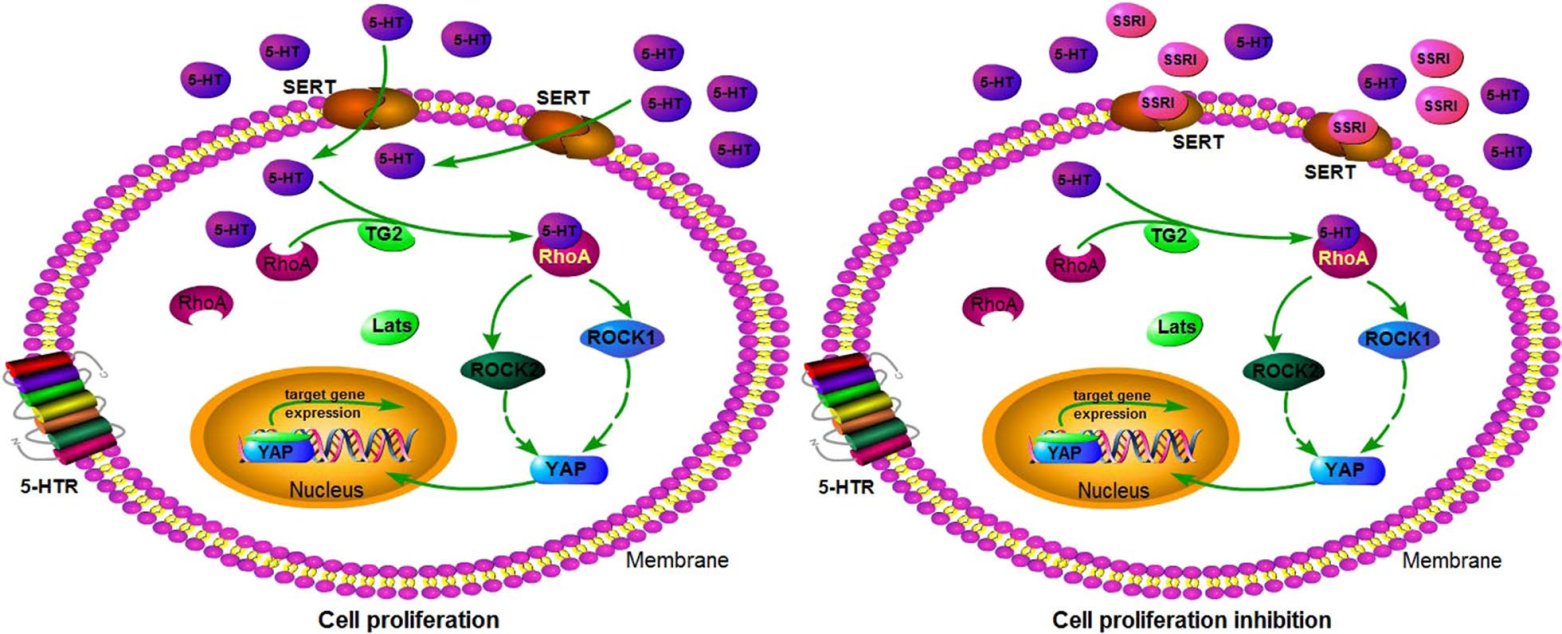
Schematic representation of the proposed mechanism by which 5-HT modulates signalling pathways in colon cancer. **A** In colon cancer cells, extracellular 5-HT is transported into cells by SERT located in the cytomembrane and then transamidates and activates RhoA via serotonylation regulated by TG2. Subsequently, ROCK1/2 is activated, leading to the expression of YAP and its target proteins. **B** When SERT function is blocked by specific antagonists (such as citalopram), 5-HT intracellular transportation is weakened to some degree, blocking the cellular signalling transduction and colon cancer cell proliferation induced by 5-HT. This strategy suggests that targeting 5-HT signalling may be feasible for treating colon cancer

In summary, we reported that SERT was responsible for transporting serotonin into colon cancer cells which TG2-mediated serotonylation to activate RhoA. This effect enhances YAP expression and promotes the carcinogenesis of colon cancer, blocking SERT by potentially impeding 5-HT’s intracellular signalling effect on cell growth (Fig. 7). These findings provided insight into serotonin’s role in promoting colorectal carcinogenesis and provided new strategies for colorectal cancer therapy.

## Supplementary Information

**Additional file 1. Table S1.** The sequences of primers for siRNA and qPCR.

**Additional file 2. Figure S1.** Subcellular localization of YAP in colon cancer cells induced by 5-HT. Subcellular localization of YAP expression in SW480 or SW1116 cells after 10 μM 5-HT stimulation for 4 h was shown by immunofluorescence (400×).

**Additional file 3. Figure S2.** GPCR has not affected YAP expression in colon cancer cells. **A** Subtypes of GPCR in SW480 and SW1116 cells were knocked down by transfecting small interfering RNAs; a western blot was used to analyse YAP and Cyr61 expression in cells treated with 10 μM 5-HT. **, *P* < 0.01. **B** Hela cells were stimulated with 10 μM 5-HT after transfection with hSERT pcDNA3 vector, and YAP expression was analysed by western blot.

**Additional file 4. Figure S3.** LC-MS/MS detected cytoplasmic 5-HT in colon cancer cells. Cytoplasmic 5-HT is observable in colon cancer cells following 5-HT stimulation. Subsequently, LC–MS/MS identified a prominent dissociative peak at 1.49 min in both SW480 and SW1116 cell lysates after 10 μM 5-HT stimulation, similar to the standard.

**Additional file 5. Figure S4.** Colocalization of 5-HT and RhoA in colon cancer cells. Immunofluorescence was performed to show that the co-expression of 5-HT and RhoA in SW480 and SW1116 cells stimulated with 10 μM 5-HT in the absence and presence of citalopram (100 μM), added 2 h before 5-HT stimulation (400×).

**Additional file 6. Figure S5.** Morphological changes of intestinal mucosa structures in mice. A HE staining showed intestinal mucosa structures in experimental mice of indicated groups (200×). B YAP expression in colorectal carcinoma tissues of indicated group of mice was analysed by immunohistochemistry (200×).

## Data Availability

All data are included in the study and available from the corresponding author on reasonable request.

## Abbreviations

5-HT: 5-Hydroxytryptamine
5-HTRs: 5-HT receptors
SERT: Serotonin transporter
SSRIs: Serotonin reuptake inhibitors
YAP: Yes-associated protein
LC–MS/MS: Liquid chromatography–tandem mass chromatography
TG2: Transglutaminase-2
ROCK: Rho-associated protein kinase
Tph1: Tryptophan hydroxylase-1
Cyr61: Cysteine-rich angiogenic inducer 61
