# Supplementary material for: Serotonin acts through YAP to promote cell proliferation: mechanism and implication in colorectal cancer progression
Source: Cell Commun Signal. 2023 Apr 12;21:75. doi: 10.1186/s12964-023-01096-2 (PMC10100184; doi:10.1186/s12964-023-01096-2)
Supplement: Supplementary file 2 — Additional file 1. Table S1. The sequences of primers for siRNA and qPCR. [file 12964_2023_1096_MOESM2_ESM.docx]

**Supplement Tab. The sequences of primers for siRNA and qPCR**

| si-YAP | GACAUCUUCUGGUCAGAGA |
| --- | --- |
| si-LATS1 | CACGGCAAGATAGCATGGATT |
| si-LATS2 | CTCCGCAAAGGGTACACTCAA |
| si-GNA12 | CUUGAGCGCCUAUGACUUG |
| si-GNA13 | GAAGAUCGACUGACCAAUC |
| si-GNAQ | GCUGGUGUAUCAGAACAUC |
| si-GNA11 | GACACCGAGAATATCCGCTTT |
| si-TG2 | AAGGGCGAACCACCTGAACAA |
| si-RhoA | CAGCUGGGGCGGAAGAUUATT |
| si-SERT | TTCACAGTGCTCGGTTACA |
| si-control | UUCUCCGAACGUGUCACGUTT |
| GNA12 forward | 5′-CTCAAGGGCTCAAGGGTTCTT-3′ |
| GNA12 reverse | 5′-CAGGAACATCCCATGCTTCTC-3′ |
| GNA13 forward | 5′-CGGAAACGCTGGTTTGAATGC-3′ |
| GNA13 reverse | 5′-AGATTCTGTAAGGCGATTGGTCT-3′ |
| GNAQ forward | 5′-CTCGAGCCACCATGACTCTGGAGTCCATCATGG-3’ |
| GNAQ reverse | 5′-GCGGCCGCTTAGACCAGATTGTACTCCTTCAG-3′ |
| GNA11 forward | 5′- CTCGAGCCACGATGACTCTGGAGTCCATGATGG-3′ |
| GNA11 reverse | 5′- GCGGCCGCTCAGACCAGGTTGTACTCCTTG-3′ |
| GAPDH forward | 5′-CAAGGCCAACCGCGAGAA-3′ |
| GAPDH reverse | 5′-CCCTCGTAGATGGGCACAGT-3′ |
