# Supplementary figures and images for: Serotonin acts through YAP to promote cell proliferation: mechanism and implication in colorectal cancer progression
Source: Cell Commun Signal. 2023 Apr 12;21:75. doi: 10.1186/s12964-023-01096-2 (PMC10100184; doi:10.1186/s12964-023-01096-2)

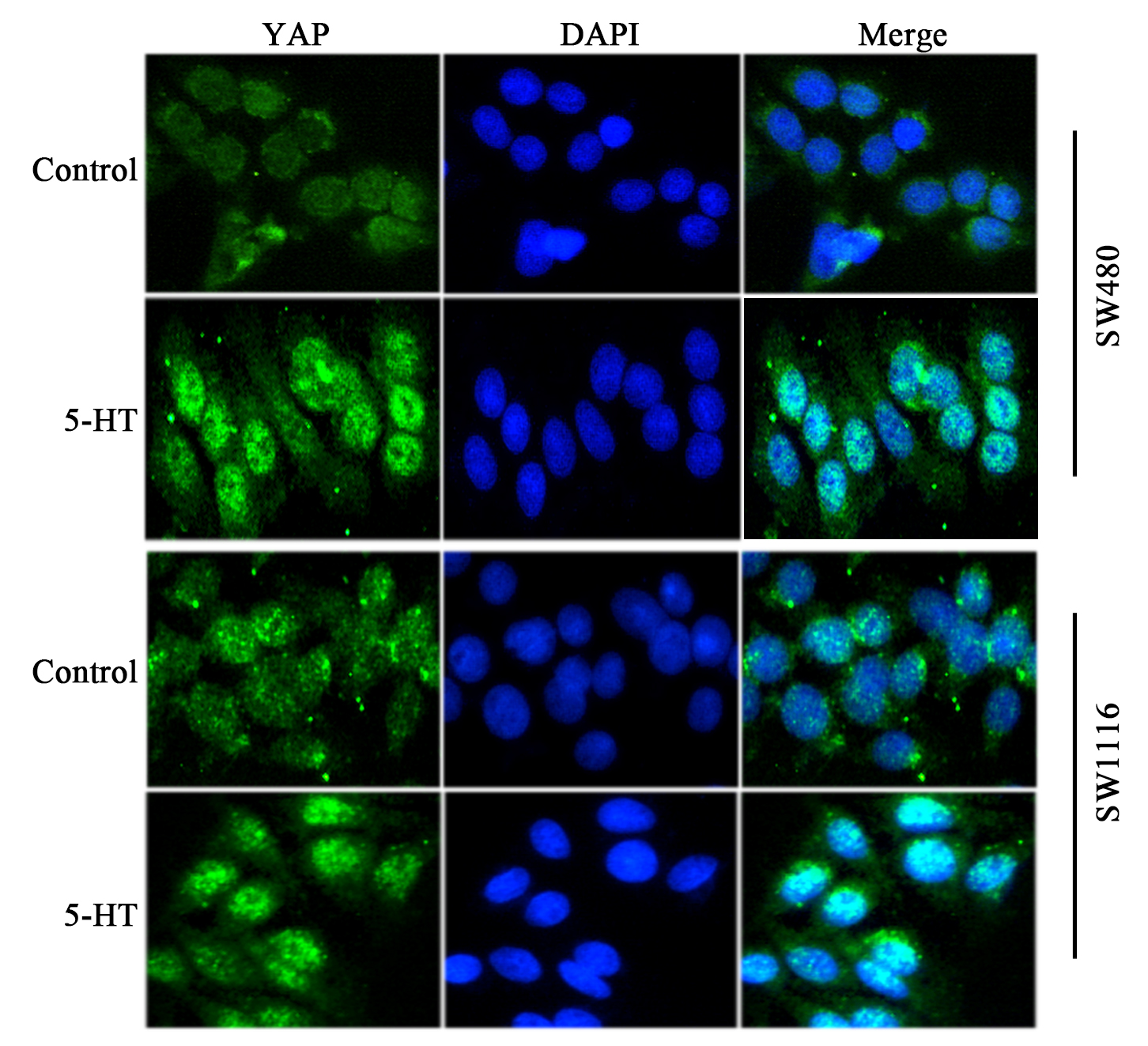

Supplement: Supplementary file 3 — Additional file 2. Figure S1. Subcellular localization of YAP in colon cancer cells induced by 5-HT. Subcellular localization of YAP expression in SW480 or SW1116 cells after 10 μM 5-HT stimulation for 4 h was shown by immunofluorescence (400×). [file 12964_2023_1096_MOESM3_ESM.jpg]

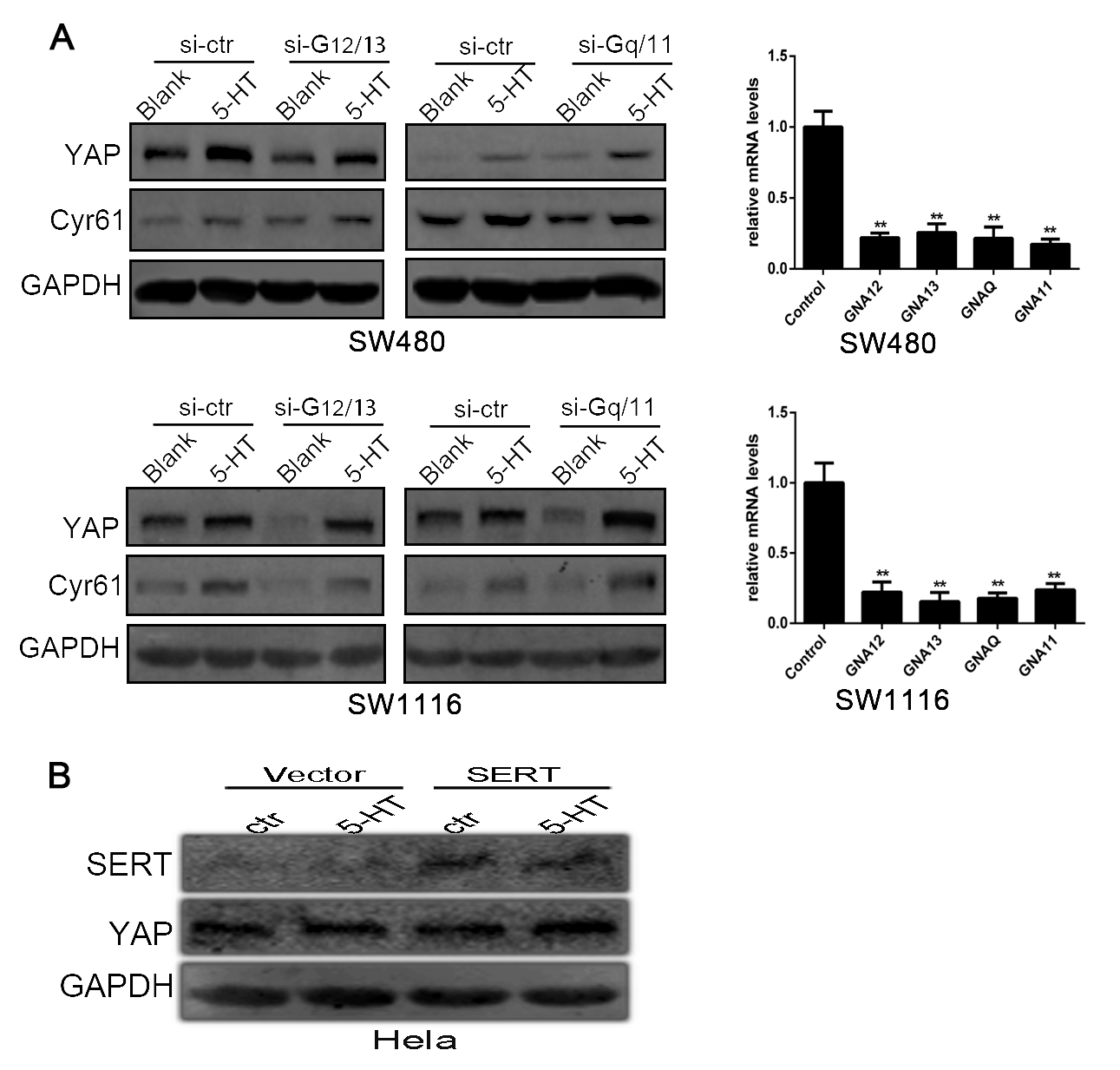

Supplement: Supplementary file 4 — Additional file 3. Figure S2. GPCR has not affected YAP expression in colon cancer cells. A Subtypes of GPCR in SW480 and SW1116 cells were knocked down by transfecting small interfering RNAs; a western blot was used to analyse YAP and Cyr61 expression in cells treated with 10 μM 5-HT. **, P < 0.01. B Hela cells were stimulated with 10 μM 5-HT after transfection with hSERT pcDNA3 vector, and YAP expression was analysed by western blot. [file 12964_2023_1096_MOESM4_ESM.tif]

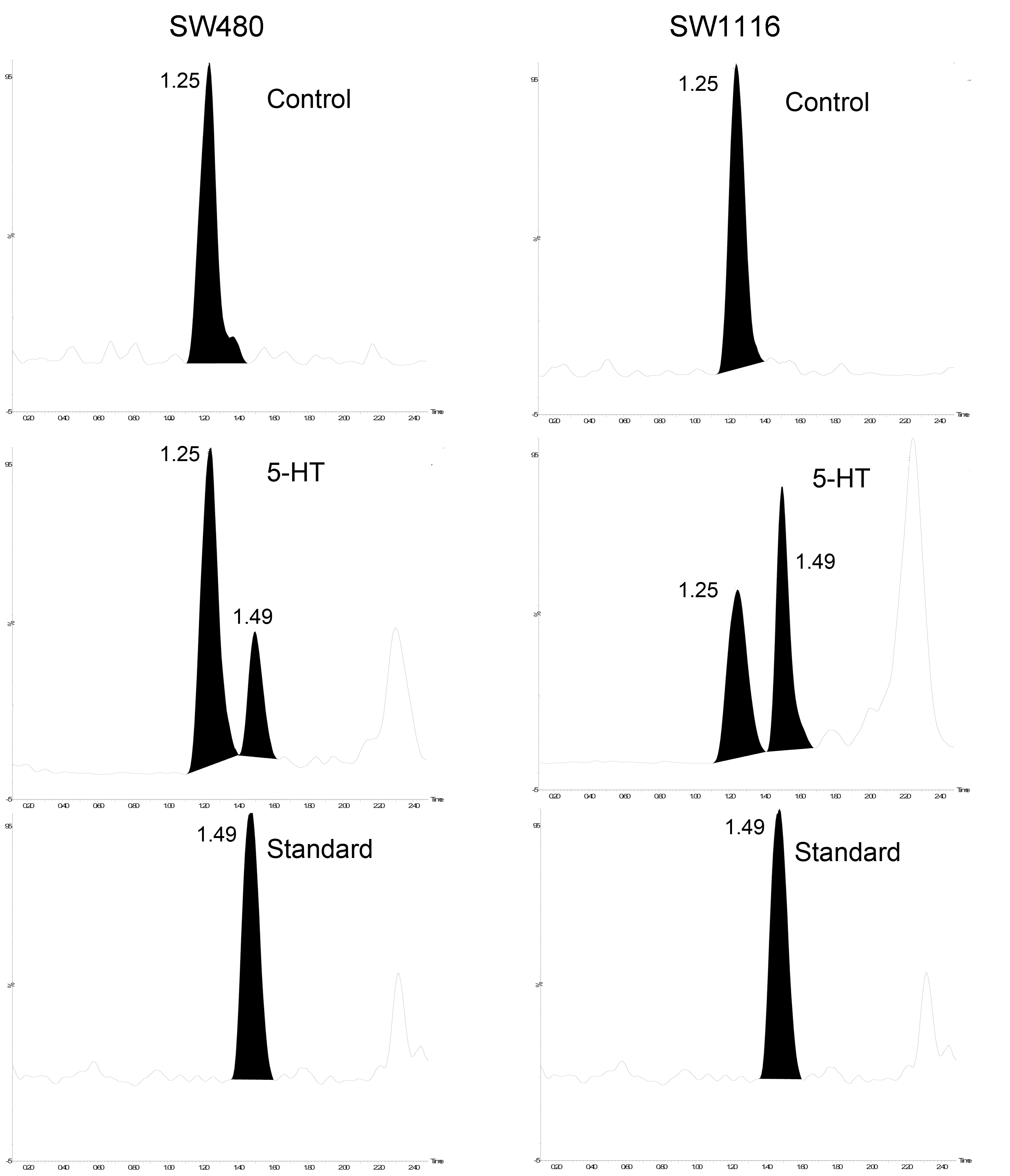

Supplement: Supplementary file 5 — Additional file 4. Figure S3. LC-MS/MS detected cytoplasmic 5-HT in colon cancer cells. Cytoplasmic 5-HT is observable in colon cancer cells following 5-HT stimulation. Subsequently, LC–MS/MS identified a prominent dissociative peak at 1.49 min in both SW480 and SW1116 cell lysates after 10 μM 5-HT stimulation, similar to the standard. [file 12964_2023_1096_MOESM5_ESM.tif]

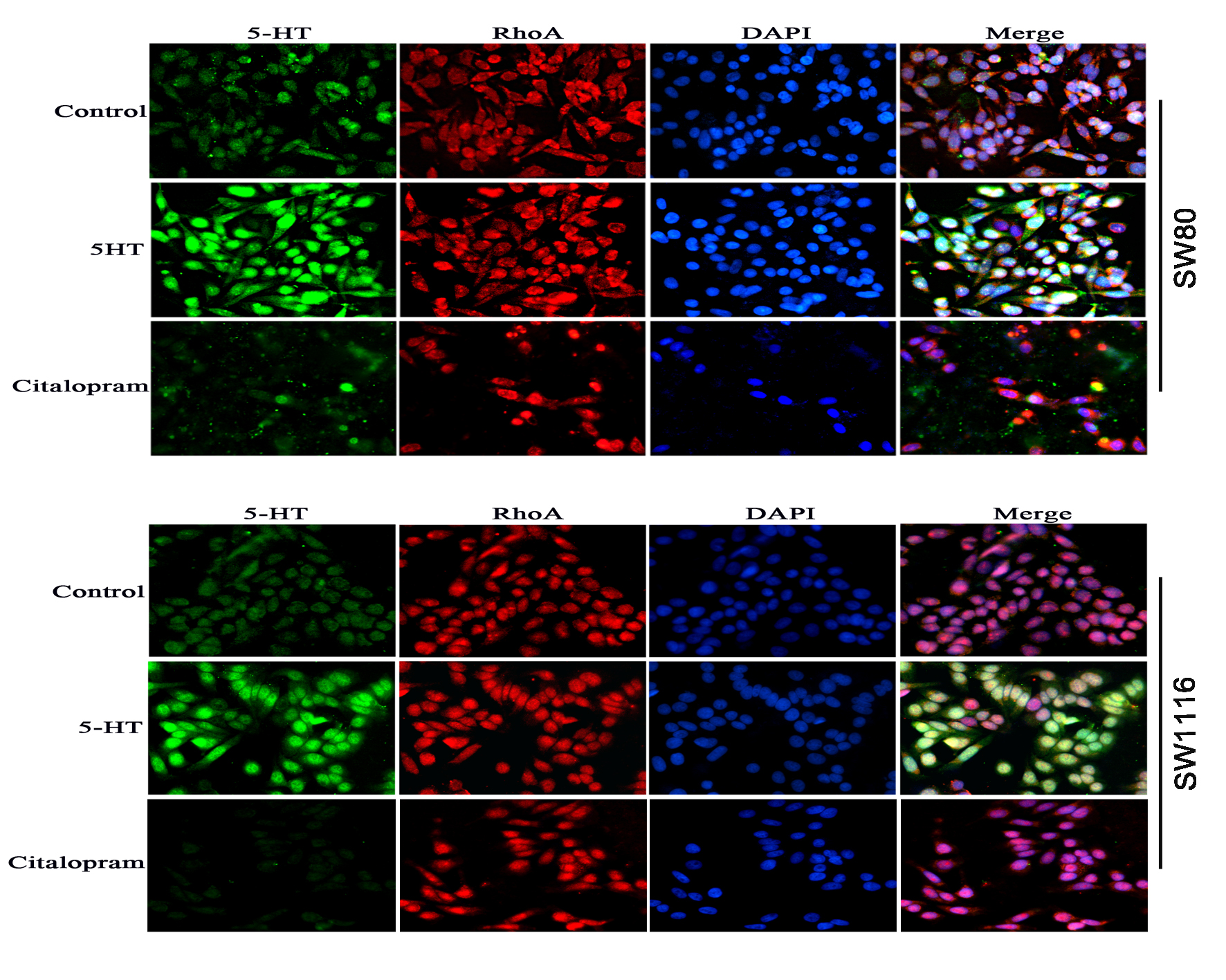

Supplement: Supplementary file 6 — Additional file 5. Figure S4. Colocalization of 5-HT and RhoA in colon cancer cells. Immunofluorescence was performed to show that the co-expression of 5-HT and RhoA in SW480 and SW1116 cells stimulated with 10 μM 5-HT in the absence and presence of citalopram (100 μM), added 2 h before 5-HT stimulation (400×). [file 12964_2023_1096_MOESM6_ESM.jpg]

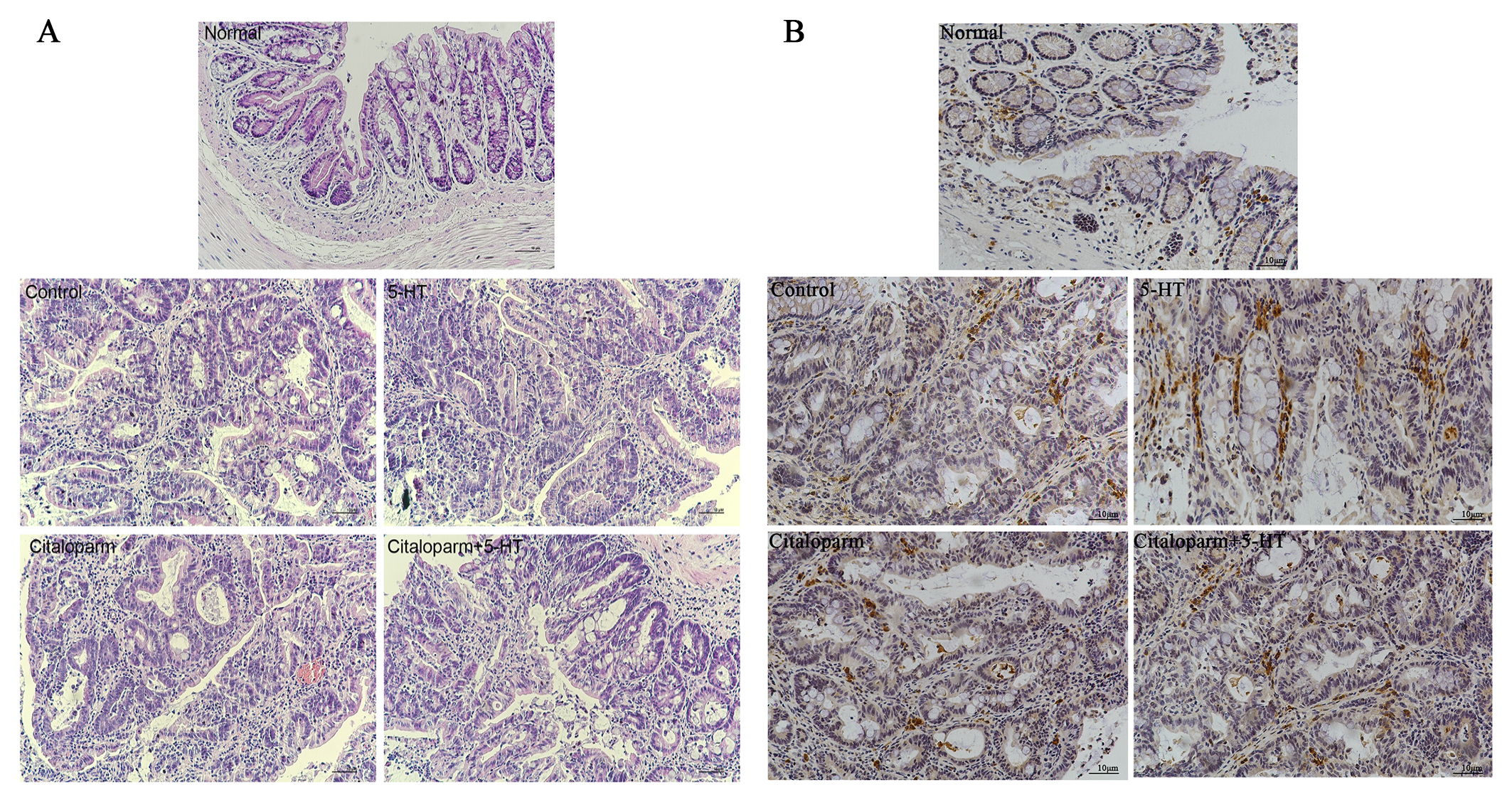

Supplement: Supplementary file 7 — Additional file 6. Figure S5. Morphological changes of intestinal mucosa structures in mice. A HE staining showed intestinal mucosa structures in experimental mice of indicated groups (200×). B YAP expression in colorectal carcinoma tissues of indicated group of mice was analysed by immunohistochemistry (200×). [file 12964_2023_1096_MOESM7_ESM.jpg]
